# The characteristics of high-quality Yb:YAG single crystal fibers grown by a LHPG method and the effects of their discoloration

**DOI:** 10.1039/c9ra03633d

**Published:** 2019-07-22

**Authors:** Tao Wang, Jian Zhang, Na Zhang, Siyuan Wang, Baiyi Wu, Zhitai Jia, Xutang Tao

**Affiliations:** State Key Laboratory of Crystal Materials, Shandong University Jinan 250100 China z.jia@sdu.edu.cn txt@sdu.edu.cn; Key Laboratory of Functional Crystal Materials and Device, Ministry of Education Jinan 250100 China

## Abstract

Single crystal fibers (SCFs), especially ytterbium (Yb) doped crystal fibers, have great potential in the field of high-power lasers. Colorless Yb:YAG single crystal fibers were fabricated using a laser heated pedestal growth (LHPG) method with a diameter fluctuation of less than 2% and a length to diameter ratio greater than 320 : 1. An abnormal color issue exists with respect to Yb:YAG crystals. The origin of coloration was studied *via* density functional theory, single-crystal X-ray diffraction, XPS and Raman spectroscopy and it was confirmed that the cyan coloration of Yb:YAG crystals is due to oxygen vacancies. Yb:YAG SCFs prepared *via* the LHPG method could avoid this type of defect due to the large specific surface area and melt convection caused by surface tension. The fundamental properties of the cyan Yb:YAG crystal source rod and colorless Yb:YAG SCFs were systematically investigated. The colorless Yb:YAG SCFs have higher infrared transmittance and thermal conductivity. The distributions of Yb^3+^ along the radial and axial directions were also measured. Meanwhile we demonstrated the propagation loss and a fiber laser using the colorless Yb:YAG SCFs, obtaining a minimum loss coefficient of 0.008 dB cm^−1^ and a maximum continuous-wave (CW) output power of 3.62 W. The colorless Yb:YAG SCFs with good thermal conductivity, low propagation loss, wide transparency and uniform ion distribution show promise for acting as the host material in single-mode lasers.

## Abstract 2

High-quality Yb:YAG single crystal fibers were grown successfully *via* LHPG method and the corresponding properties were investigated in detail.

## Graphical abstract

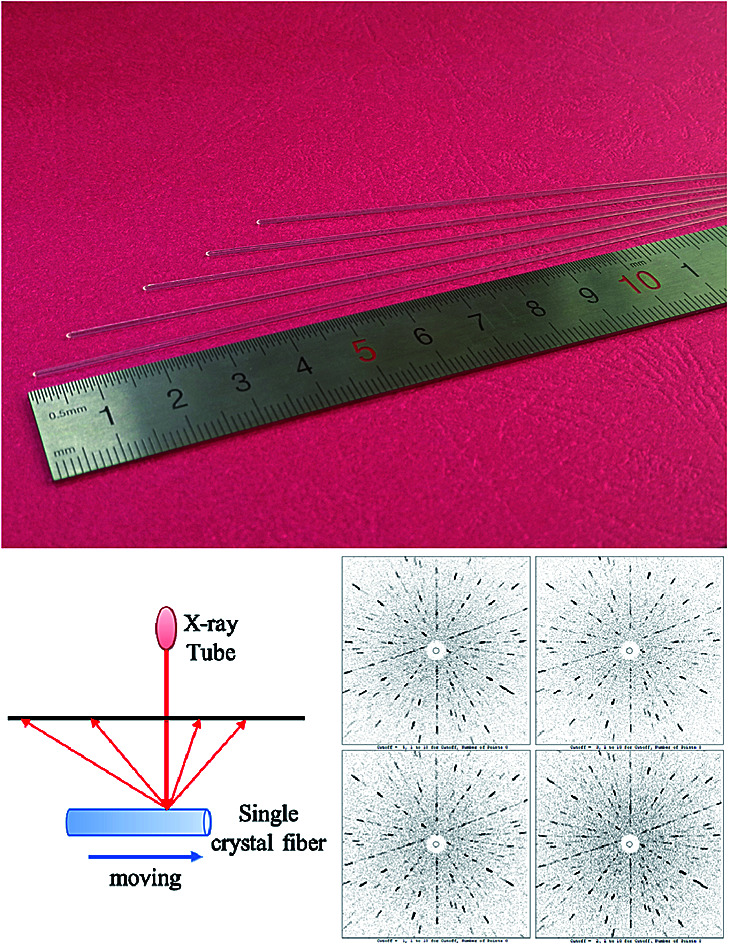

## 1. Introduction

Nowadays, with the explosive development of solid-state lasers, high-power lasers are gradually being applied to materials processing, information and communication technology, medical treatment and many other fields. Therefore, increasing the output power and reducing thermal effects present huge challenges for traditional solid-state gain media, such as glass, ceramics and crystals.^1–8^ Because of their excellent optical conversion efficiency, good beam quality and lower maintenance cost, fiber lasers are gradually becoming a new hotspot in the field of solid-state lasers. Their output power has reached 10 kW in continuous-wave form, mainly in ship-borne lasers and laser weapons. Compared with traditional solid-state laser media, fibers have a larger specific surface area, which greatly improves the thermal management of the laser. However, due to limitations inherent in the material properties, it is difficult to obtain higher output powers with conventional glass fibers. Therefore, crystal fibers with higher thermal conductivity, better mechanical properties and higher damage thresholds can be used to promote the development of next-generation high-power lasers.^9–17^

Yttrium aluminium garnet (YAG) belongs to a cubic system, with the unit cell parameter *a* = 12.0089 Å. YAG crystals have good physical and chemical stabilities and have been widely used in solid-state lasers.^18^ At the same time, YAG crystals can achieve high doping concentrations because of the similar sizes of yttrium atoms and rare-earth atoms. Compared to traditional silica fibers, YAG crystal fibers have a lower Brillouin gain coefficient, a higher laser damage threshold and higher thermal conductivity. The theoretical single-mode output of a YAG SCF laser is more than 100 times that of a glass fiber laser.^19–23^

Yb-doped YAG crystals are an excellent laser medium. Yb^3+^ ions have a simple electronic structure, with only one excited state (2F_5/2_) above the ground state (2F_7/2_), avoiding thermal effects due to up-conversion and excited state absorption. Moreover, the theoretical segregation coefficient of Yb^3+^ is close to 1, which is beneficial for miniaturizing devices. Compared to Nd^3+^, Yb^3+^ is well suited for IR InGaAs diode laser pumping between 900 and 980 nm. Meanwhile, the close absorption (900–980 nm) and emission wavelengths (980–1100 nm) lead to a low thermal load (11% relative to 30% in Nd^3+^-doped laser hosts), thereby significantly improving the slope efficiency, which reaches almost 90% theoretically.^24–26^ Therefore, Yb:YAG crystals are considered to be the most promising fiber material for 1 μm band lasers.^27–30^ A continuous-wave output power of 251 W obtained by a Yb:YAG SCF laser is also reported to be a record in the field of crystal fiber lasers.^31^

However, it is interesting to find that commercial Yb:YAG crystals are usually different shades of cyan, and the color always deepens with an increase in the crystal size. This color issue relating to Yb:YAG crystals should be the primary problem to be solved, especially if they are going to be used in high-power lasers. Usually, most commercial Yb:YAG crystals are grown *via* the Czochralski method. An inert or reductive atmosphere may be the cause of the discoloration.^32,33^ This color issue has puzzled people for a long time, but the origin of the coloration of Yb:YAG crystals has never been systematically justified.

In this work, we have successfully fabricated colorless and transparent Yb:YAG SCFs using Yb:YAG bulk crystals with a cyan color in both oxygen-rich and inert atmospheres *via* a LHPG method. Besides, we have designed an experimental scheme to seek the origins of the coloration. Some optical and thermal properties of cyan Yb:YAG crystal source rods and colorless YAG SCFs were also compared. The second part of this paper relates to the fundamental characteristics of Yb:YAG SCFs, including diameter fluctuations, crystal quality, propagation loss and ion distribution. A Yb:YAG fiber laser is also reported in this paper.

## 2. Experimental

### 2.1 Growth of single crystal fibers

**Fig. 1:**
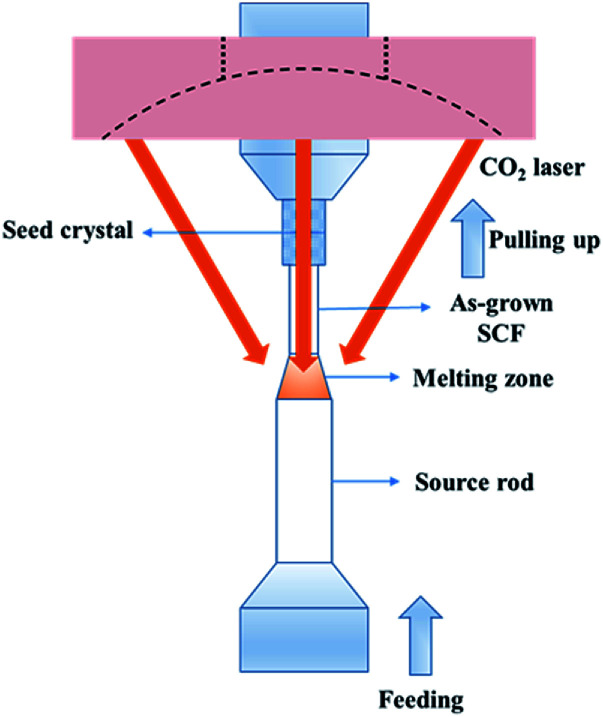
A schematic diagram of the LHPG technique.

A schematic diagram detailing the LHPG method is shown in Fig. 1. Generally, solid-phase sintered ceramic or bulk crystals are used as the source rods and seed crystals. We used a 2% Yb-doped YAG bulk crystal as the source rod; moreover, an undoped YAG crystal orientated in the 〈111〉 direction was selected as the seed crystal. The ring CO_2_ laser is focused on the top of the source rod to form a melting zone. The seed crystal is immersed in the melting zone and kept at the center of the source rod to stable the melting zone. Approximately 50 to 60 W of laser power is needed to melt a 3 mm-diameter source rod. The growth rate is about 0.2–1 mm min^−1^ which means one hundred millimeters of fiber can be obtained in 2 h. The ratio of the pulling speed to the feeding speed determines the diameter of the crystal fiber; generally, this is no more than 9 : 1, converting to a diameter ratio of 3 : 1. Therefore, some SCFs need to be grown twice to obtain finer examples. Compared with other crystal growth methods, there is no crucible in the LHPG method, which greatly improves the purity of the raw materials. At the same time, non-crucible growth conditions enrich the growing atmosphere. We can use not only inert atmospheres but also air or even oxygen atmospheres during crystal growth.

**Fig. 2:**
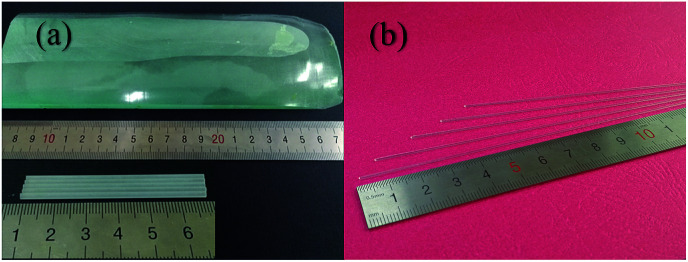
(a) The Yb:YAG bulk crystal and processed source rods. (b) As-grown Yb:YAG SCFs.

As shown in Fig. 2, the Yb:YAG crystal source rods are cyan, however, after processing *via* the LHPG method, colorless and transparent crystal fibers were obtained without any post-processing. In this paper, we obtained *Φ*2.0 × 50 mm^3^ Yb:YAG SCFs with a pulling ratio of 3 : 1; these were then grown twice, obtaining *Φ*0.8 × 260 mm^3^ and *Φ*0.6 × 150 mm^3^ Yb:YAG SCFs with pulling ratios of 5 : 1 and 9 : 1, and a length-to-diameter ratio greater than 320 : 1.

### 2.2 Single-crystal X-ray diffraction

In order to compare the cell parameters of the cyan source rod and the colorless single crystal fiber, single-crystal X-ray diffraction studies were performed using D8 VENTURE apparatus and the patterns were obtained using a Bruker SMART APEX-III diffractometer equipped with a CCD area detector and using graphite-monochromated Mo-Kα radiation (*λ* = 0.71073 Å) at 296 K. Diffraction data collection and unit-cell refinement were performed using the INTE-GRATE program with APEX-3 software.

### 2.3 X-ray photoelectron spectroscopy

XPS measurements were carried out to verify the existence of O^2−^ ions using Thermo ESCALAB 250XI apparatus. The tested samples were wafers of the cyan source rod and as-grown colorless SCFs.

### 2.4 Optical properties

The UV-visible extinction spectra of the Yb:YAG crystal source rod and as-grown Yb:YAG SCFs were recorded over a wavelength range extending from 200 to 900 nm using a Cary 50 spectrophotometer.

The RT optical transmission spectra of the Yb:YAG crystal source rod and as-grown Yb:YAG SCFs were obtained using a PE IR SPECTRUM ASCII PEDS 1.60 Fourier transform infrared (FTIR) spectrometer over the wavenumber range of 450–7800 cm^−1^.

The RT Raman spectra of the Yb:YAG crystal source rod and as-grown Yb:YAG SCFs were recorded using a Horiba Jobin Yvon LabRAM HR Raman spectrometer equipped with a liquid N_2_ cooled Ge detector. A solid laser power of 150 mW at 532 nm was used for excitation.

### 2.5 Thermal properties

The specific heats of the Yb:YAG crystal source rod and as-grown Yb:YAG SCFs were measured using a differential scanning calorimeter (DSC-Q20) from 30 to 295 °C at a heating rate of 5 °C min^−1^.

The thermal conductivities were measured using the Thermal Transport Option (P670B) of a Quantum Design Physical Property Measurement System (PPMS) from 4–400 K with a liquid nitrogen cooling system. The cooling and heating rates selected in the experiment were 6 °C min^−1^ and 0.5 °C min^−1^, respectively.

### 2.6 Computational details

**Fig. 3:**
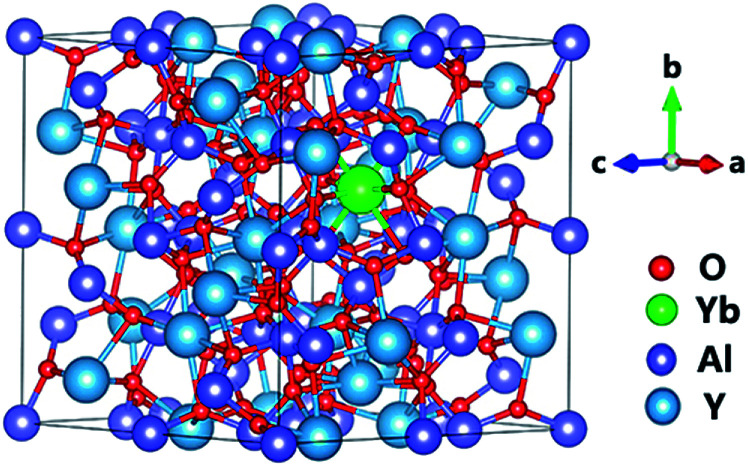
The optimized Yb:YAG crystal structure. The red, green, purple, and blue spheres represent O, Yb, Al, and Y atoms, respectively.

Geometry optimization calculations were carried out on Yb:YAG based on density functional theory (DFT) with the Perdew–Burke–Ernzerhof (PBE) generalized gradient approximation (GGA) functional for the exchange correlation function, implemented using the Vienna Ab initio Simulation Package (VASP).^34–36^ The energy cutoff was set to 450 eV, and the structure relaxation convergence threshold was set to 10^−5^ eV in energy terms and 0.02 eV Å^−1^ in force terms. A 2 in2 in2 Monkhorst–Pack mesh for Brillouin zone integration was used during geometry calculations. The structure of Yb:YAG is shown in Fig. 3. In order to explore whether the existence of Yb^3+^ will change the oxygen vacancy formation trend, we compared the formation energies of oxygen vacancies at different Yb^3+^ doping concentrations. The absorption spectra and refractive indices under different defect conditions were also simulated.

### 2.7 Diameter fluctuation

Diameter fluctuation is one of the most important parameters for evaluating the quality of single crystal fibers, as it reflects the stability of the crystal growth process. In this paper, the diameter fluctuations of Yb:YAG SCFs were characterized using an LS-9000 laser micrometer. SCFs with diameters of 0.6 mm and 0.8 mm were selected as the samples. In order to improve the accuracy of the experiments, we took measurements every millimeter and repeated measurements 100 times for each sample.

### 2.8 High-resolution X-ray diffraction (HRXRD)

HRXRD was performed using a Bruker D8-discover diffractometer equipped with a four-crystal Ge(220) monochromator set for Cu-Kα radiation (*λ* = 1.54056 Å). The accelerating voltage and tube current of the generator were 40 kV and 20 mA, respectively. The step time and step size were set to 0.5 s and 0.001°, respectively. A double-sided, polished, (111)-oriented Yb:YAG SCF wafer was used as the HRXRD sample with dimensions of *Φ*0.8 × 1.0 mm^3^.

### 2.9 Laue back-reflection measurements

To further determine the crystallinity of the SCFs, Laue back-reflection measurements were carried out using a real-time back-reflection Laue camera system (Multiwire MWL 120 with Northstar software) to further assess the crystalline perfection of the Yb:YAG SCFs. A (111)-oriented YAG SCF with dimensions of *Φ*0.6 × 50 mm^3^ was employed for the Laue diffraction measurements.

### 2.10 Electron probe microanalysis (EPMA)

The distributions of Yb^3+^ along the radial and axial directions were characterized using EPMA-1720H apparatus (Shimadzu, Japan). The accelerating voltage and tube current of the generator were 15 kV and 100 nA, respectively. The distributions were measured along three diameter directions and the radial direction. 300 points were selected for each set of experiments and they were measured for 0.5 s per point. A double-sided polished Yb:YAG SCF was used as the EPMA sample with dimensions of *Φ*0.6 × 5.0 mm^3^.

### 2.11 Propagation loss

**Fig. 4:**
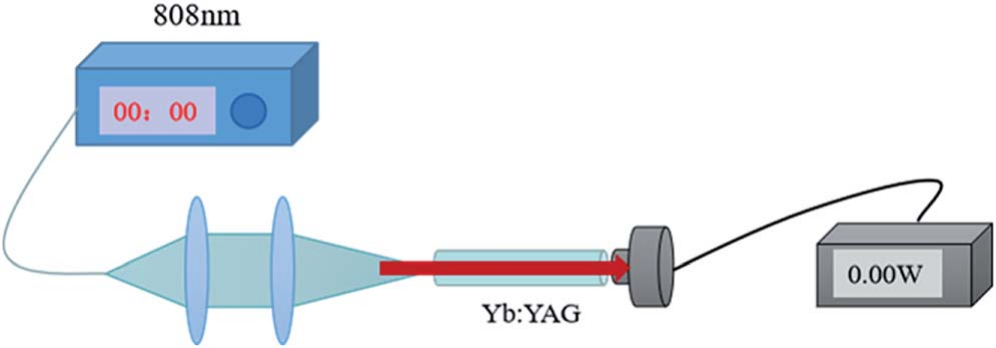
A simplified schematic diagram of the propagation loss test set-up.

The propagation loss of a Yb:YAG SCF was measured *via* a cut-back method. A schematic diagram of the propagation loss experiment set-up is shown in Fig. 4. The light source used in the test is a fiber-coupled 808 nm semiconductor laser, which can effectively avoid loss caused by spectral absorption, improving the accuracy of the test. In order to make the test more accurate, different lengths of fibers were selected for testing.

### 2.12 Laser experiments

The emission spectra of as-grown Yb:YAG SCFs were obtained employing an FLS-980 spectrometer (Edinburgh Instruments).

**Fig. 5:**
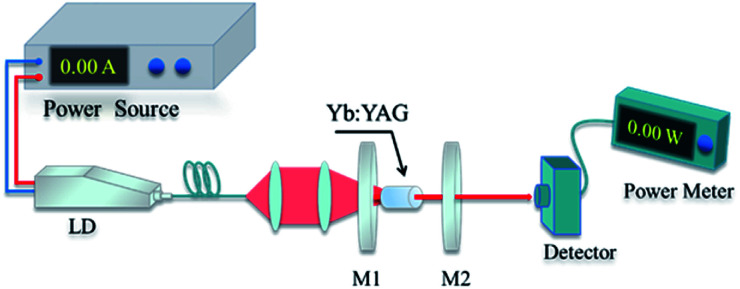
A schematic diagram of the Yb:YAG SCF laser setup.

The experimental apparatus used for the laser experiments is depicted in Fig. 5. A Yb:YAG SCF was pumped using a fiber-coupled 940 nm semiconductor laser with a 200 μm core diameter and a NA of 0.22. The magnification ratio of the focusing system is 1 : 1. The pump beam was focused to a 200 μm diameter, with the focal point located on the input side of the SCF. The input mirror (IM) is a concave mirror, which was coated for high transmission (*T* > 98%) at the pump wavelength (940–976 nm) and high reflectivity (*R* > 99%) from 1020 to 1060 nm. The output mirror (OM) was plated with 1020–1070 nm partially reflective films with transmittances of 3%, 10% and 30%, respectively. The IM was placed as close as possible to the input side of the SCF, so as to provide as much feedback as possible at the lasing wavelength. The resonant cavity consisted of a flat-concave short cavity structure with a length of 11 mm. In order to reduce the thermal effects of the crystal, the SCF was placed in a copper block and the temperature was set to 15 °C. Spectral detection was performed using an AQ6370D spectrum analyzer and the scanning interval was set to 1020 nm to 1080 nm to facilitate observations. *Φ*0.6 × 10 mm^3^ Yb:YAG SCFs (2 at% doping) were applied in the laser experiments.

## 3. Results and discussion

### 3.1 Unit-cell parameters

The phenomenon of discoloration occurring in oxide crystals is generally considered to be caused by oxygen defects. In order to determine the type of oxygen deficiency, we collected the unit cell parameters of the cyan source rod and colorless SCFs grown under an inert atmosphere. As shown in Table 1, the cell parameters (*a* = *b* = *c* = 12.03 Å) and cell volume (1743 Å^3^) of the source rod are smaller than those of the colorless SCFs (*a* = *b* = *c* = 12.05 Å, *V* = 1749 Å^3^). We can speculate from these results that the discoloration of the source rod may be caused by vacancies.

**Table 1:** The unit cell parameters of the source rod and as-grown SCFs

	*a* (Å)	*b* (Å)	*c* (Å)	*V* (Å^3^)
Yb:YAG source rod	12.03	12.03	12.03	1743
As-grown Yb:YAG SCFs	12.05	12.05	12.05	1749

### 3.2 XPS studies

**Fig. 6:**
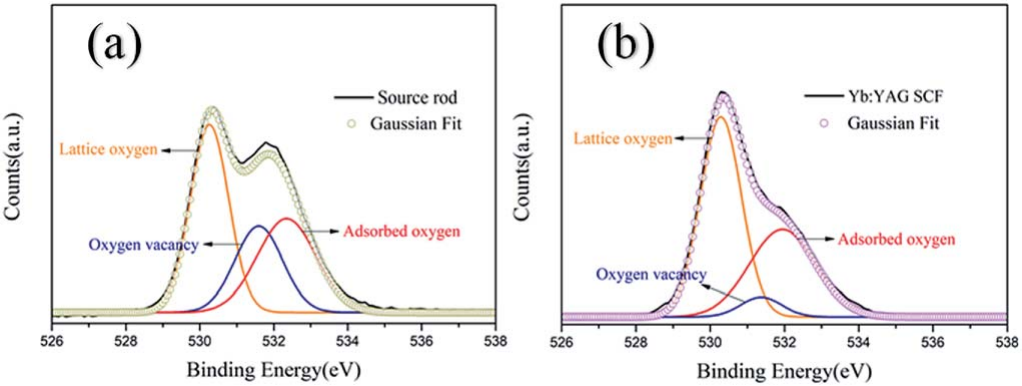
O1s XPS spectra of (a) the source rod, and (b) as-grown Yb:YAG SCFs.

Fig. 6 shows O1s XPS spectra from the Yb:YAG crystal source rod and as-grown Yb:YAG SCFs under an inert atmosphere. The spectra are decomposed into peaks using the Gaussian model. There are three Gaussian peaks at 530.23 eV, 531.41 eV and 532.40 eV. Peak 1 (at 530.23 eV) represents the lattice oxygen ion content. Peak 2 (at 531.41 eV) is generally considered to be caused by oxygen vacancies. Peak 3 (at 532.40 eV) is caused by adsorbed water and adsorbed oxygen.^37^ It can be seen from the figure that the content of oxygen vacancies (peak 2) in the colorless SCFs is lower than that in the source rod, further confirming that the color is related to oxygen vacancies. The large specific surface area of the SCFs and the melt convection caused by surface tension make it possible to avoid the generation of oxygen vacancies, even in an inert environment.

### 3.3 Optical properties

**Fig. 7:**
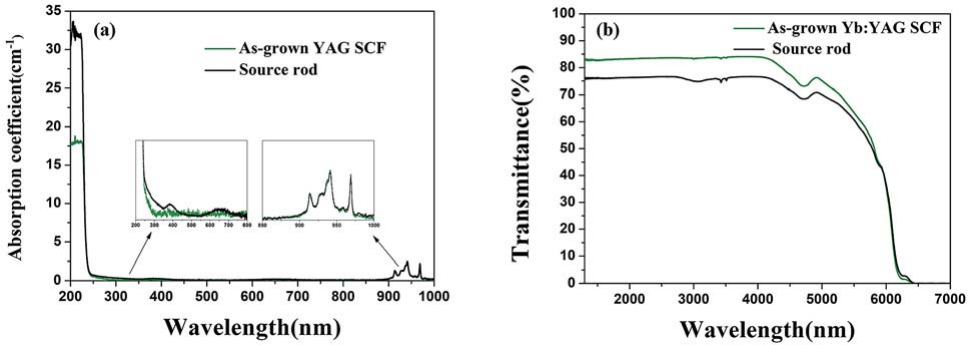
(a) UV-visible extinction spectra and (b) infrared transmission spectra.

UV-visible extinction spectra of the Yb:YAG crystal source rod and the as-grown Yb:YAG SCFs under an inert atmosphere are shown in Fig. 7(a). We can see that the Yb^3+^ characteristic peaks of the two samples are consistent, and the absorption coefficients are basically the same. However, the source rod has absorption peaks at 380 nm and 650 nm. Optical transmission spectra of the source rod and as-grown Yb:YAG SCFs under an inert atmosphere were recorded over the wavelength range of 1300–8000 nm, as shown in Fig. 7(b). We found that the colorless Yb:YAG SCFs exhibit higher infrared transmittance.

**Fig. 8:**
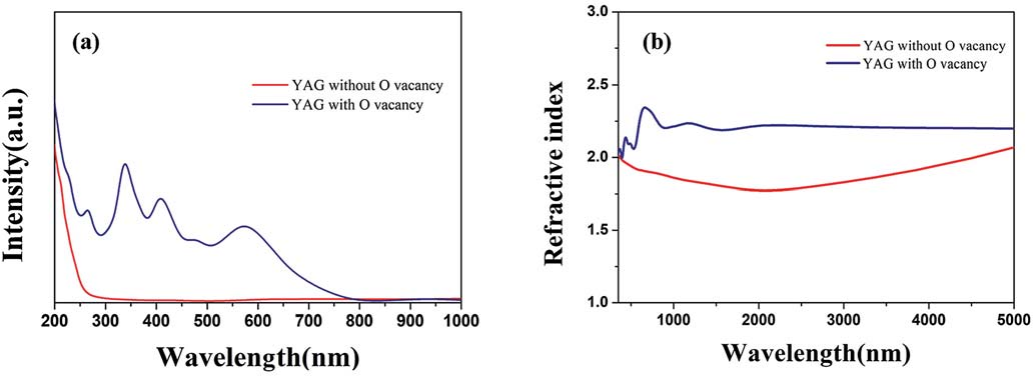
Calculated (a) absorption spectra and (b) refractive index values under different defect conditions.

Fig. 8 shows simulated absorption spectra and refractive indices under different defect conditions. From the simulation results in Fig. 8(a), we can find that YAG crystals with oxygen vacancies show obvious absorption in the region of 300–650 nm, which is consistent with the measured absorption spectrum of the discolored Yb:YAG crystal. Fig. 8(b) shows that the Yb:YAG crystal is predicted to possess a higher refractive index in the presence of oxygen vacancies. The Fresnel reflection is positively correlated with differences in the refractive indices at the interface. Therefore, a higher refractive index caused by oxygen vacancies in the Yb:YAG crystal will increase the reflectance at the interface, resulting in greater optical loss. In the measured infrared transmission spectra shown in Fig. 7(b), the transmittance of the discolored sample is lower compared with a colorless sample of the same thickness, which is consistent with the simulation results.

**Fig. 9:**
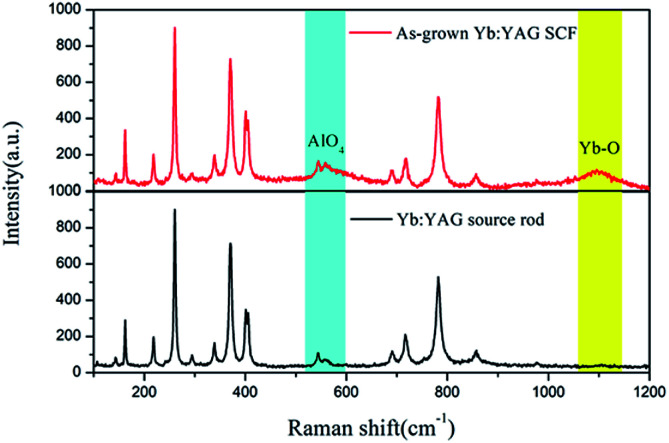
Raman scattering spectra of the source rod and as-grown Yb:YAG SCFs.

Raman spectra of the source rod and the as-grown SCFs under an inert atmosphere are shown in Fig. 9. By comparison, we found that the intensity of the Raman peak at 546 cm^−1^ has decreased for the cyan source rod sample and the Raman peak at 1100 cm^−1^ has disappeared. These two peaks are considered to be attributed to the υ_2_ mode of AlO_4_ units and Yb–O vibrations, respectively.^38^ These data indicate that the formation of oxygen vacancies in Yb:YAG is related to Yb^3+^.

### 3.4 Thermal properties

Thermal properties are important for laser crystals, of which thermal conductivity is critical for achieving high energy laser applications.

**Fig. 10:**
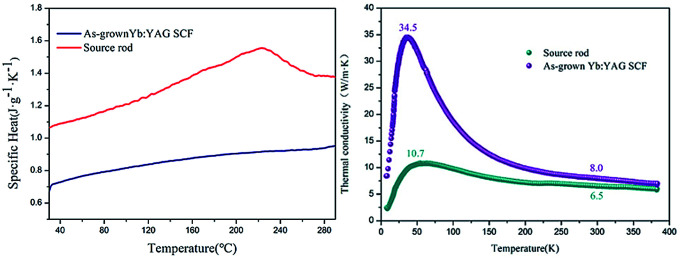
Thermal properties: (left) specific heat and (right) thermal conductivity as a function of temperature.

The specific heat (*C*_v_) values of the Yb:YAG crystal source rod and the colorless Yb:YAG SCFs grown under an inert atmosphere as a function of temperature are shown in Fig. 10. As can be seen, the specific heat of the Yb:YAG SCF increases from 0.679 J g^−1^ K^−1^ at 30 °C to 0.973 J g^−1^ K^−1^ at 295 °C. The specific heat of the source rod is 1.0653 J g^−1^ K^−1^ at 30 °C; as the temperature increases, it increases to 1.3723 J g^−1^ K^−1^ at 295 °C. Lattice thermal vibration is considered to be one of the important factors affecting the specific heat of crystalline materials. The formation of oxygen vacancies will increase the lattice distortion, which is considered to cause abnormal fluctuations in the specific heat of the source rod.

The thermal conductivity (*κ*) of the crystals can be calculated using the following formula:1
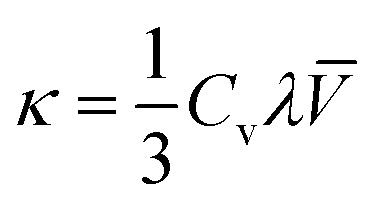
where *C*_v_, *λ* and *V̄* denote the specific heat of the crystal, the mean free path of phonons and the average speed of phonons, respectively. Thermal conductivity *versus* temperature plots (4–383 K) of the source rod and as-grown Yb:YAG SCFs under an inert atmosphere are shown in Fig. 10. The thermal conductivity of the Yb:YAG SCF along the 〈111〉 direction reaches a maximum of 34.5 W m^−1^ K^−1^ at 40 K. As the temperature increases, the thermal conductivity gradually approaches 7.1 W m^−1^ K^−1^ (at 100 °C). The thermal conductivity of the cyan source rod was significantly lower than that of the colorless crystal fiber at the same temperatures, especially in low temperature environments. The thermal conductivity at low temperatures is mainly determined by the mean free path of phonons. However, the presence of defects, impurities, and grain boundaries will greatly reduce the mean free path of phonons, which explains why the cyan source rod has lower thermal conductivity. The lower thermal conductivity will reduce the thermal management abilities of the fiber laser and increase thermal effects inside the fiber, thus affecting the laser power and beam quality.

### 3.5 Formation energy of oxygen vacancies

Calculations of the formation energy of oxygen vacancies are carried out in accordance with the following formula:2
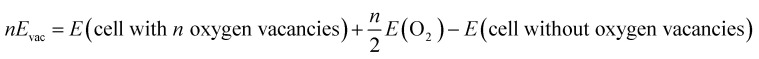


Table 2 lists the free energy values of the system under different conditions and the oxygen vacancy formation energies. The formation energy of an oxygen vacancy is 6.51 eV in a YAG crystal. As the Yb^3+^ content increases to 4% and 8%, the formation energies of oxygen vacancies decrease to 6.48 eV and 6.06 eV, respectively. We speculate that this is related to the valence state of Yb^3+^. When Yb^3+^ is converted to Yb^2+^, a negative electric center is formed, which induces positive electric vacancies around it. Therefore, choosing an appropriate doping concentration is also an effective way to avoid defects.

**Table 2:** Oxygen vacancy formation energies of Yb:YAG under different conditions

Type	*E*_tot_/eV	*E*_vac_/eV
4O_2_	−33.63	
Y_24_Al_40_O_96_, without oxygen vacancies	−1303.06	
Y_24_Al_40_O_88_, with 8 oxygen vacancies in dispersed states	−1217.32	6.51
Y_23_Yb_1_Al_40_O_96_, without oxygen vacancies	−1298.68	
Y_23_Yb_1_Al_40_O_88_, with 8 oxygen vacancies	−1213.20	6.48
Y_22_Yb_2_Al_40_O_96_, without oxygen vacancies	−1290.45	
Y_22_Yb_2_Al_40_O_88_, with 8 oxygen vacancies	−1208.27	6.07

### 3.6 Diameter fluctuation

One of the most serious difficulties faced in relation to oxide single crystal fibers is controlling the fiber diameter. The situation is quite different from glass fibers; because SCFs are drawn from high temperature melt, any vibrations or irregular motion during growth can lead to diameter fluctuation.

Movement instability (fiber or source rod) and power fluctuations of the carbon dioxide laser are the main reasons for diameter fluctuations in fibers, especially for micron-sized single crystal fibers.

**Fig. 11:**
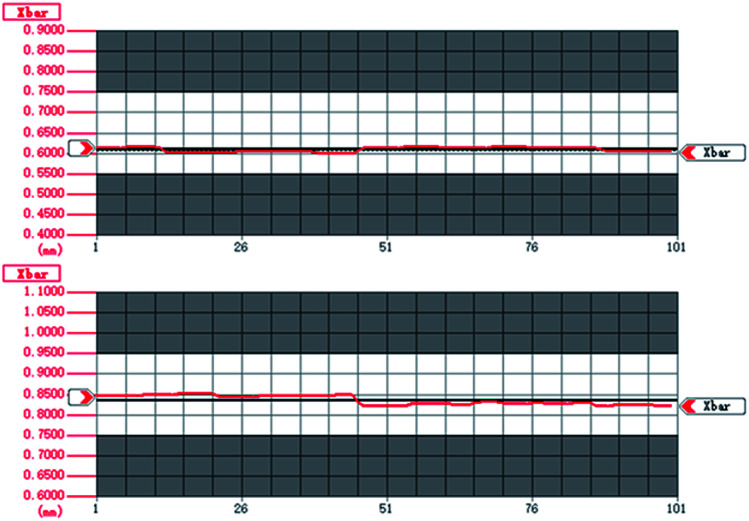
The diameter fluctuations of Yb-doped YAG SCFs: (top) *Φ*600 μm; (bottom) *Φ*800 μm.

Through the adjustment of growth parameters and the optimization of the laser, the diameter stability of the SCFs has been obviously improved. The diameter fluctuations of two YAG SCFs are shown in Fig. 11. The average diameters of the two samples are 0.6089 mm and 0.8352 mm, respectively. At the same time, the data indicates that the diameter ranges (the difference between the maximum and minimum diameters) of the two YAG SCFs are 0.02 mm and 0.03 mm, representing excellent diameter control of better than ±2%, which is beneficial for the fabrication of cladding and related devices.

**Fig. 12:**
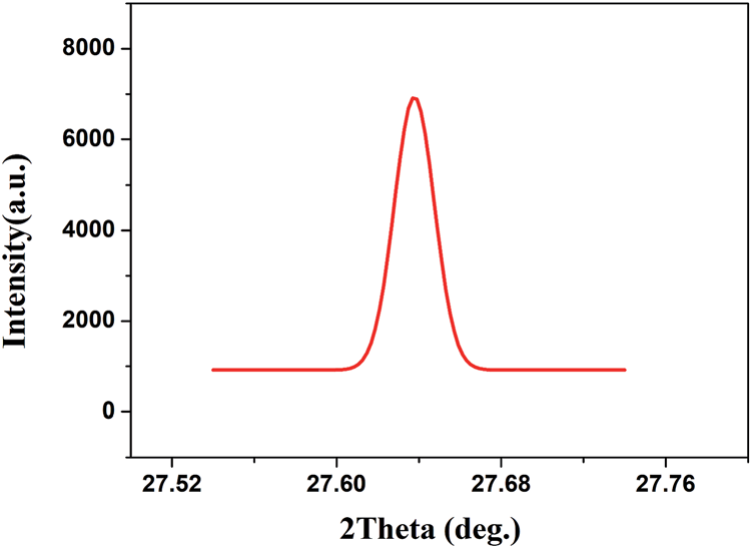
X-ray rocking curve of the (111) diffraction plane of the Yb:YAG SCFs.

**Fig. 13:**
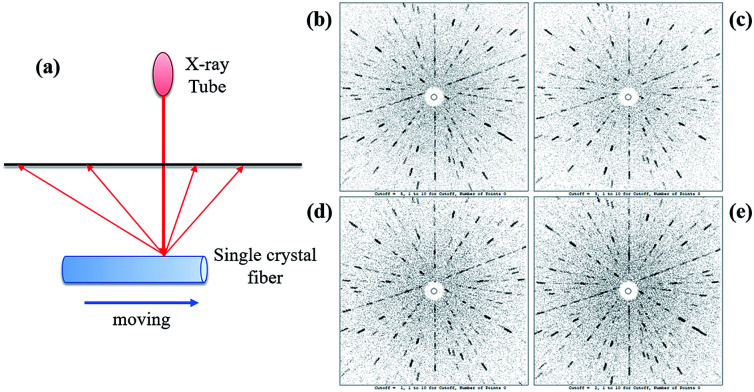
(a) A schematic diagram showing the positions of the Laue back-reflection measurements on the crystal fiber. (b–e) Characteristic Laue back-reflection patterns at different positions with the X-ray beam hitting the crystal fiber.

The quality of the as-grown Yb:YAG SCFs was characterized *via* HRXRD and Laue back-reflection measurements. The rocking curve of the (111) diffraction plane is shown in Fig. 12. The diffraction peak presents a symmetric shape without splitting. The full width at half maximum (FWHM) is estimated to be 72.2′′. Laue back-reflection measurements are broadly applied to investigate the crystallinity and orientation of an as-grown crystal. As can be apparently seen from Fig. 13, the characteristic Laue back-reflection patterns at different positions along the crystal fiber are uniform, clear, and bright. The above results demonstrate that the crystalline quality of the Yb:YAG SCFs is high enough, which furnishes the basis for assessing their intrinsic physical properties.

### 3.7 Distribution of Yb^3+^

**Fig. 14:**
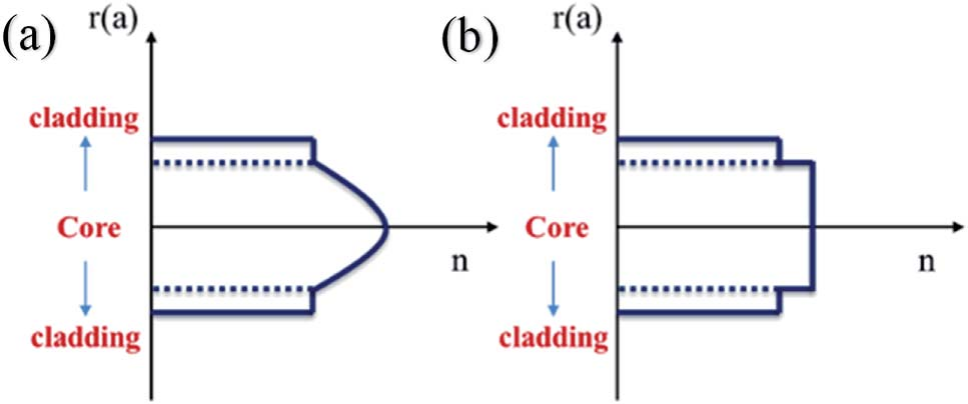
The refractive index of a SCF: (a) gradual type and (b) mutant type.

The refractive index of the fiber can be expressed *via* the following equation:3
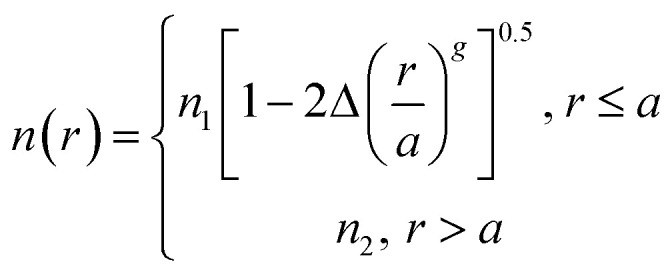
where *n*_1_ and *n*_2_, represent the refractive indices of the core and cladding, respectively, *Δ* is the relative refractive index difference, *r* and a are the radial coordinates and core radius, respectively, and *g* stands for the distribution index of the refractive index. Due to differences in the value of *g*, the distribution of the refractive index can be divided into “gradual type” and “mutant type”, as shown in Fig. 14.

In order to achieve single-mode laser output, a fiber must maintain a mutant refractive index profile, which means that the refractive index of the core must be uniform. For doped crystals, it is necessary to ensure the uniformity of rare earth ion distribution. We determined the elemental compositions of the source rod and crystal fibers *via* X-ray fluorescence (XRF). As shown in Table 3, the concentrations of Yb^3+^ in the source rod and the as-grown SCF were measured to be 2.36 and 2.35, respectively. The calculated segregation coefficient of Yb^3+^ is close to 1.

**Table 3:** Elemental compositions of the source rod and an as-grown SCF

	Al (mass%)	Y (mass%)	Yb (mass%)	Yb/Y (at%)
Source rod	21.2	75.3	3.46	2.36
As-grown SCF	25.5	72.1	3.31	2.35

**Fig. 15:**
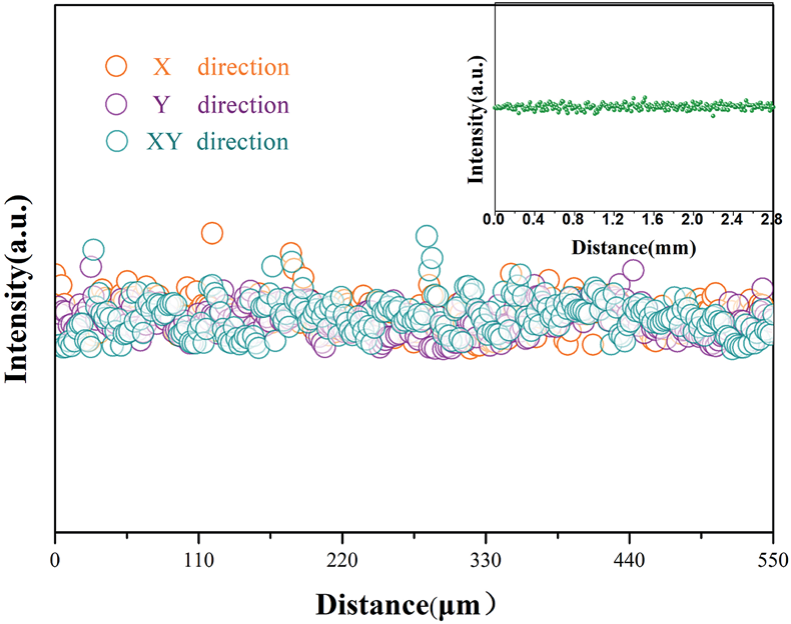
The radial distribution of Yb^3+^. The inset shows the distribution of Yb^3+^ along the axial direction.

The concentrations of Yb^3+^ along the radial and axial directions are shown in Fig. 15. We found that Yb^3+^ is uniformly distributed in both the radial and axial directions without significant ion-rich regions, which is a key factor for achieving single-mode lasers.

### 3.8 Propagation loss

The following standard formulas were used to calculate the attenuation coefficients on the log scale:4
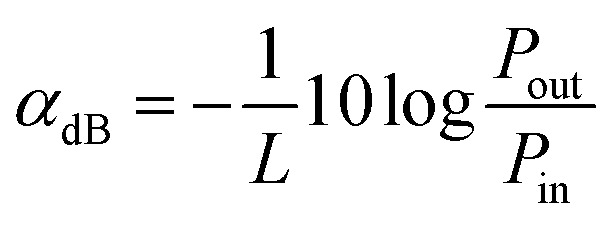
where *L*, *P*_out_, and *P*_in_ denote the fiber length, the output laser power, and the input laser power, respectively. Fresnel reflection loss is included in the total fiber loss. Therefore, both ends of the fiber are polished to laser quality and the test results were also corrected for Fresnel reflections. We selected two parts of different lengths from the same fiber; the Fresnel reflection losses and the attenuation coefficients of the two samples are considered to be equal at the same power, thus eliminating any error caused by Fresnel reflections. The obtained measurement results are presented in Table 4. We found that the propagation attenuation coefficient is around 0.01 dB cm^−1^, indicating that the grown crystal fibers are of good quality and possess excellent optical waveguiding performance.

**Table 4:** Attenuation coefficients at different powers

Sample	*L* (cm)	*P*_in_ (W)	*P*_out_ (W)	*P*_out_/*P*_in_ (without subtraction of the reflection loss)	*P*_out_/*P*_in_ (with subtraction of the reflection loss)	Reflection loss	*α*_dB_
Fiber 1	0.8	1.034	0.914	88.39%	99.76%	11.37%	0.013 dB cm^−1^
Fiber 2	1.8	1.034	0.911	88.10%	99.47%	11.37%	0.013 dB cm^−1^
Fiber 1	0.8	2.003	1.763	88.02%	99.72%	11.7%	0.015 dB cm^−1^
Fiber 2	1.8	2.003	1.756	87.67%	99.37%	11.7%	0.015 dB cm^−1^
Fiber 1	0.8	3.016	2.637	87.43%	99.84%	12.41%	0.008 dB cm^−1^
Fiber 2	1.8	3.016	2.631	87.23%	99.64%	12.41%	0.008 dB cm^−1^
Fiber 1	0.8	4.005	3.490	87.14%	99.78%	12.64%	0.012 dB cm^−1^
Fiber 2	1.8	4.005	3.479	86.87%	99.51%	12.64%	0.012 dB cm^−1^

### 3.9 Lasing with a Yb:YAG SCF

**Fig. 16:**
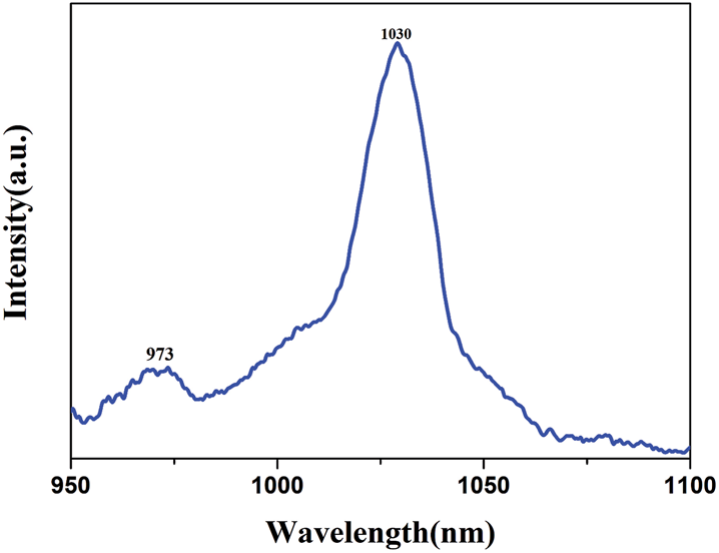
The emission spectrum of a Yb:YAG SCF.

**Fig. 17:**
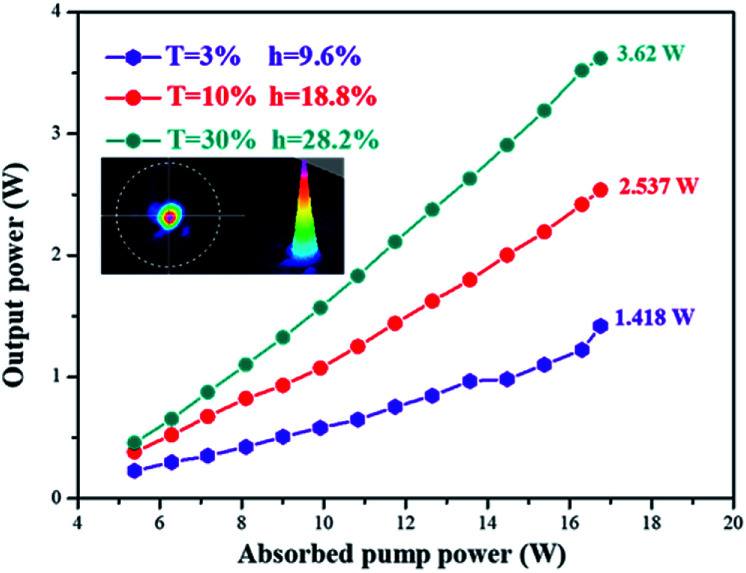
The output laser power as a function of absorbed pump power with different output mirrors.

The laser wavelength is centered at 1030.82 nm, which is consistent with the emission spectrum (shown in Fig. 16). Fig. 17 shows the output laser power as a function of the absorbed pump power under CW pumping. A maximum output power of 3.62 W was measured for an output mirror with transmittance of 30% at an absorbed pump power of 16.75 W. Taking into account all the Fresnel reflection losses and the un-absorbed pump power, the slope efficiency was calculated to be 28.2% with a lasing threshold of 3.92 W. The output beam satisfies a Gaussian distribution and the beam quality factor *M*^2^ was measured to be 1.45, which could be further optimized by using a more compact resonator.

## 4. Conclusions

In this paper, colorless Yb:YAG SCFs with a length-to-diameter ratio exceeding 320 : 1 were successfully grown in both aerobic and anaerobic environments *via* a LHPG method. The diameter fluctuation is less than 2%, indicating that the crystal growth process is stable and the crystal fibers are relatively uniform. By means of single crystal X-ray diffraction, XPS and Raman spectroscopy, we found that the colorless Yb:YAG SCFs possess larger unit cell parameters, fewer oxygen vacancies and more obvious AlO_4_ and Yb–O vibrations than the cyan Yb:YAG crystal source rod. Therefore, we conclude that the discoloration of Yb:YAG crystals is caused by oxygen vacancies, and the formation energy of oxygen vacancies decreases as the Yb^3+^ content increases. We believe that the large specific surface area of the grown Yb:YAG SCFs, the low Yb^3+^ doping concentration and the surface tension convection of the LHPG method are the main factors in avoiding defects.

At the same time, some optical and thermal properties of the cyan Yb:YAG crystal source rod and colorless Yb:YAG SCFs were measured. The discolored Yb:YAG crystal, with discoloration caused by oxygen vacancies, has lower infrared transmittance and thermal conductivity, which will reduce the laser output and the beam quality dramatically. Meanwhile, the as-grown SCFs were confirmed to be of high quality from rocking curve and Laue back-reflection measurements, and the propagation attenuation coefficient is around 0.01 dB cm^−1^. Through EPMA measurements, we found that Yb^3+^ is uniformly distributed in the YAG SCFs, which is necessary for achieving single-mode lasers. Based on this, we carried out laser experiments under different conditions and achieved a maximum continuous-wave (CW) output power of 3.62 W with a slope efficiency of 28.2%. In conclusion, all properties, including the small diameter fluctuation, good crystal quality, good thermal conductivity and uniform ion distribution, prove that the colorless Yb:YAG SCFs are a promising host material for single-mode lasers.

## Conflicts of interest

There are no conflicts to declare.

## Supplementary Material
